# Comparative transcriptomes reveal differential effects on host metabolism reprogramming in two different pelagiphage-SAR11 infection systems

**DOI:** 10.1093/ismeco/ycaf233

**Published:** 2025-12-08

**Authors:** Zefeng Zhang, Xinxin Liu, Yahui Zhang, Hang Xiao, Pei Liu, Mingyu Yang, Fang Qin, Ying Wu, Hanqi Ying, Zuqing Wu, Yanlin Zhao

**Affiliations:** College of Life Sciences, Fujian Agriculture and Forestry University, Fuzhou, Fujian 350002, China; College of Life Sciences, Fujian Agriculture and Forestry University, Fuzhou, Fujian 350002, China; College of Life Sciences, Fujian Agriculture and Forestry University, Fuzhou, Fujian 350002, China; College of Life Sciences, Fujian Agriculture and Forestry University, Fuzhou, Fujian 350002, China; College of Life Sciences, Fujian Agriculture and Forestry University, Fuzhou, Fujian 350002, China; College of Life Sciences, Fujian Agriculture and Forestry University, Fuzhou, Fujian 350002, China; College of Life Sciences, Fujian Agriculture and Forestry University, Fuzhou, Fujian 350002, China; College of Life Sciences, Fujian Agriculture and Forestry University, Fuzhou, Fujian 350002, China; College of Life Sciences, Fujian Agriculture and Forestry University, Fuzhou, Fujian 350002, China; College of Life Sciences, Fujian Agriculture and Forestry University, Fuzhou, Fujian 350002, China; College of Life Sciences, Fujian Agriculture and Forestry University, Fuzhou, Fujian 350002, China; Key Laboratory of Marine Biotechnology of Fujian Province, Institute of Oceanology, Fujian Agriculture and Forestry University, Fuzhou, Fujian 350002, China

**Keywords:** SAR11, pelagiphage, phage-host interaction, RNA-seq, transcriptome

## Abstract

As the most abundant marine microorganisms, SAR11 bacteria contribute significantly to global carbon and nutrient cycling. Pelagiphages, viruses that infect SAR11, are potential drivers in structuring the communities, shaping the evolution, and reprogramming the metabolism of SAR11. However, interactions between SAR11 and pelagiphages remain poorly understood. In this study, we investigated and compared the transcriptional dynamics of the SAR11 strain, *Candidatus* Pelagibacter communis HTCC1062, under independent infection with two phylogenetically distinct pelagiphages: the temperate HTVC019P-type pelagiphage HTVC022P and the lytic HTVC023P-type pelagiphage HTVC027P. These two pelagiphages exhibited distinct infection kinetics, with HTVC022P showing a shorter latent period and a faster host takeover. Transcriptome profiling revealed that infection with HTVC022P and HTVC027P led to the differential expression of 136 and 460 host genes, respectively. Compared to the uninfected control, both pelagiphage infections enhanced host transcription, upregulating the majority of differentially expressed genes. Both pelagiphages induced upregulation of host genes involved in DNA metabolism, transcription, translation, central carbon and nitrogen metabolism. Notably, HTVC027P infection led to the upregulation of 56 genes involved in phosphate, sulfur, and iron metabolism, as well as oxidative phosphorylation and one-carbon metabolism. In contrast, HTVC022P had minimal effects on these pathways. These results suggest that distinct pelagiphages exert unique effects on host metabolic processes, implying divergent ecological implications. Collectively, our study provides new insights into SAR11-pelagiphage interactions, enhancing our understanding of the metabolic states of phage-infected SAR11 bacteria and the ecological functions of phages in marine systems.

## Introduction

Viruses are the most abundant and diverse biological entities in the ocean, and marine viral communities primarily comprise phages that infect bacteria [1–5]. Through diverse phage-host interactions, phages can alter the bacterial community structure, metabolic activity, and evolutionary trajectories, thereby profoundly influencing marine biogeochemical processes [1–3, 6, 7]. Phage-infected cells, termed virocells, exhibit physiological and metabolic characteristics distinct from uninfected cells [8–10]. Despite the ecological significance of phages and the predominance of virocells in the ocean, our understanding of host metabolic reprogramming following phage infection remains limited.

Genome-wide transcriptome sequencing throughout the infection process enables a detailed understanding of the temporal dynamics of phage-host interactions. Recent transcriptome studies in several marine phage-host model systems have revealed the complex and dynamic interactions between phages and their hosts [11–17]. These studies demonstrated that phage genes are modularized and sequentially expressed during infection, with those related to host takeover, DNA replication, virion assembly, and maturation expressed in a stepwise manner [11–13, 15–19]. Concurrently, many host genes related to nucleotide metabolism, amino acid metabolism, and DNA replication were reported to be upregulated in most phage-host models, suggesting the hijacking of host cellular machinery by phages to ensure progeny replication [11, 13, 16, 17, 19]. In addition, phages were found to reprogram host metabolic mechanisms during infection. For example, in some phage-host models, genes related to central carbon, phosphorus, nitrogen, and energy metabolism were differentially expressed [11, 13, 16–18, 20, 21]. Moreover, phages can regulate host metabolism by expressing phage-encoded auxiliary metabolic genes (AMGs), which may enhance specific host metabolic processes and thus improve host survival during infection [17, 18, 21–26]. These studies demonstrate that viral infection alters the transcriptomic profile of host cells, allowing for a better assessment of the impact of phages and virocells on marine ecosystems.

The SAR11 clade (*Pelagibacterales* order) in the *Alphaproteobacteria* comprises a group of closely related free-living oligotrophic bacteria [27, 28]. SAR11 is the most abundant planktonic bacterial group in the ocean, accounting for ~25% of all marine plankton and representing ~12% of all marine prokaryotic biomass [28–30]. SAR11 bacteria possess one of the smallest genomes among all plankton in the ocean (1.28–1.49 Mbp) [28, 31]. Owing to genome streamlining and the loss of adaptive genes, SAR11 bacteria have unique carbon and nutrient requirements [28, 32, 33]. For example, the SAR11 strain, *Candidatus* Pelagibacter communis HTCC1062, requires reduced organosulfur compounds, as well as glycine or serine for growth [32, 34, 35]. In addition to monosaccharides, low-molecular-weight organic acids also serve as important carbon sources for HTCC1062 [33, 36]. HTCC1062 cells possess numerous transporters for low-molecular-weight organic compounds, and these transporters are maintained at high levels of expression and activity [28, 37]. In addition, SAR11 bacteria can oxidize a variety of one-carbon compounds, methylamines, and methylated organic compounds to generate cellular energy, participating in the regulation of carbon cycling in the ocean [38, 39]. The ecological success of SAR11 is attributed to its ability to metabolize a variety of low-molecular-weight compounds and to minimizes the resources required for replication [28, 40]. Overall, SAR11 dominates the global marine ecosystem and is a major consumer of organic carbon and sulfides in the ocean, giving it unparalleled ecological significance [28, 30, 32–34, 38, 39, 41].

The genetic diversity, life strategies, and ecological distribution of phages that infect SAR11 bacteria (pelagiphages) have been extensively investigated [42–48]. To date, over 50 pelagiphages representing eight distinct phage groups have been reported [42–49]. Metagenomic analyses revealed that pelagiphages are prevalent and dominate marine viral communities, suggesting that SAR11 cells are frequently infected by diverse pelagiphages and that pelagiphage–SAR11 interactions are the major types of phage–host interactions in the ocean [42–45]. Despite this progress, SAR11-pelagiphage interactions during infection remain poorly understood.

In this study, to assess the effects of pelagiphage infection on the metabolic states of SAR11 cells, two model systems were constructed using HTCC1062 cells and two previously isolated pelagiphages, HTVC022P and HTVC027P. The two pelagiphages are ubiquitously distributed in the ocean, representing two of the most frequently observed and ecologically significant marine bacteria-phage interactions [43]. HTVC022P and HTVC027P have different genomic compositions, representing the HTVC019P-type and HTVC023P-type groups, respectively [43, 46]. The HTVC023P-type is the most abundant known pelagiphage group in the ocean, accounting for ~7.59% of total viromic reads and outnumbering the second most abundant HTVC010P-type group by >3.6-fold [43]. The HTVC019P-type group is also prevalent, ranking as the fourth most abundant known pelagiphage group [43]. Its members belong to the *Autographivirales* and exhibit a typical T7-like genomic architecture [46]. In terms of the lifecycle, HTVC022P encodes an integrase that mediates the integration of its genome into the host tRNA-Cys site, suggesting that it also possesses a lysogenic infection cycle [46]. In contrast, HTVC027P is an obligately lytic phage that lacks genes associated with lysogeny [43]. Biogeographic analysis has revealed that both phages are widely distributed globally, with HTVC027P exhibiting a higher relative abundance across global ocean samples [43]. We determined the whole transcriptome using RNA sequencing (RNA-Seq) to elucidate the gene expression pattern throughout the infection period and to assess the effect of these pelagiphages on host transcription. The gene expression profiles of the two pelagiphages revealed the conservation of phage infection dynamics. Furthermore, our results showed that HTVC027P induced differential expression in 460 host genes, significantly more than the 136 genes affected by HTVC022P, indicating a more pronounced impact on host metabolism. HTVC027P markedly altered the transport and metabolic processes of key elements, including phosphate, sulfur, and iron metabolism. In contrast, HTVC022P had minimal impact on these pathways, with few or no differentially expressed genes (DEGs) detected in these pathways.

## Materials and methods

### SAR11 strain cultivation

The SAR11 strain, *Candidatus* Pelagibacter communis HTCC1062, was kindly provided by Prof. Stephen Giovannoni, Oregon State University. HTCC1062 cells were cultured in artificial seawater-based ASM1 medium amended with 50 μM glycine, 50 μM methionine, 100 μM pyruvate, 1 mM NH_4_Cl, 100 μM KH_2_PO_4_, and 1 μM FeCl_3_ [33]. HTCC1062 cultures were incubated at 20°C in the dark without shaking. The growth of HTCC1062 was monitored using flow cytometry as described previously [50]. Briefly, 200 μL of cultures were stained with 4× SYBR Green I (Invitrogen, Eugene, OR, USA) for 1 h in the dark. The stained cells were subsequently counted using the Guava EasyCyte (Millipore, Guava Technologies).

### Transcriptomic experiment

Pelagiphages HTVC022P and HTVC027P (GenBank accession numbers MH598798 and MN698241), were used in this study. Each phage was propagated by adding it to exponentially growing HTCC1062 cultures (~4 × 10^7^ cells/mL). The co-cultures were incubated at 20°C in the dark without shaking. HTCC1062 cell mortality was monitored using a Guava EasyCyte flow cytometer as described above. When lysis was complete, the viral lysate was collected and filtered through 0.1 μm pore size, 47 mm polycarbonate membrane filters (Isopore™, Millipore). The filtered lysate was then centrifuged using an Optima MAX-TL ultracentrifuge (Beckman Coulter, USA) at 50 000 × g for 2 h at 4°C to pellet the phage particles. The resulting pellet was then resuspended in 40 mL fresh medium and centrifuged again at 50 000 × g for 2 h at 4°C. The transcriptomic experiment was conducted in triplicate, with phage-free cultures as the control [12, 51–57]. Two-liter cultures of exponentially growing HTCC1062 cells (~4 × 10^7^ cells/mL) were independently infected with each phage at a phage-to-host ratio of ~10:1. The high phage-host ratio ensured synchronous infection of the majority of host cells. Samples for RNA extraction (200 mL) were collected at seven time points (1, 2, 3, 6, 9, 12, and 15 h post-infection (hpi)). To collect cells, samples were filtered through 0.2 μm pore size, 47 mm diameter polycarbonate membrane filters. The filters were then washed twice with 200 mL ASM1 medium through filtration to remove the remaining extracellular phages and nucleic acids. After washing, the filter membrane was placed in a 1.5 mL nuclease-free tube and flash frozen in liquid nitrogen to terminate transcription, followed by storage at −80°C.

Phage concentration during infection was quantified by real-time quantitative PCR (qPCR) with specific primers (Table S1). The relative abundance of phage DNA was determined using the comparative ΔC_T_ (threshold cycle) method, normalized against the initial phage inoculum concentration (0 h) [58]. The relative abundance of phage DNA was then calculated as ${2}^{-\Delta{\mathrm{C}}_{\mathrm{T}}}$, representing the fold change relative to the initial phage inoculum concentration. For intracellular phage quantification, 5 mL samples were collected and filtered using a 0.2 μm pore size, 25 mm diameter polycarbonate membrane (Isopore™, Millipore). The filters were washed twice with 10 mL ASM1 medium to remove extracellular phages and nucleic acids, transferred into 1.5 ml nuclease-free tubes, and immediately frozen in liquid nitrogen. Before qPCR, 1 mL of nuclease-free water was added to each tube, followed by incubation at 100°C for 10 min to lyse the cells and release intracellular DNA. For extracellular phage quantification, 1 mL of culture was collected and filtered into a 1.5 mL nuclease-free tube through a 0.1 μm pore size, 25 mm diameter acrodisc syringe filter (Pall, PAL-4611), and then frozen in liquid nitrogen. All samples were stored at −80°C until further analysis.

### RNA extraction and sequencing

Total RNA was extracted using the Direct-Zol™ RNA MiniPrep Kit (Zymo Research). The NEBNext® Ultra™ Directional RNA Library Prep Kit for Illumina (New England Biolabs) was used to construct the RNA-Seq library. The RNA-Seq library was sequenced on an Illumina HiSeq 2500 platform using a paired-end, 2 × 150 bp sequencing approach at Beijing Novogene Technology (Beijing, China). The sequencing data generated per sample were >15 Gb.

### Analysis of RNA-Seq data

SortMeRNA v4.3.4 (--fastx --out2 --paired_out) [59] was used to remove rRNA from the raw transcriptomic data, and FASTP v0.20.1 (-q 20 -l 50 -w 12) [60] was used to remove sequencing adapters, low-quality sequences with phred quality score <20, and short sequences <50 bp. These qualitatively filtered rRNA-depleted sequences were matched to the HTCC1062 reference genome and each pelagiphage genome using HISAT2 v2.2.1 (--dta -x -U -S), respectively. FeatureCounts v2.0.1 (-p -t exon -g gene_id) [61] was used to determine the number of sequence fragments mapped to each gene.

Gene expression was evaluated using normalized transcripts per million (TPM). Differential expression analysis of HTCC1062 cells was performed as previously described [12, 51–57]. Briefly, uninfected host cells at 0 h were used as controls for all time points. The differential expression of HTCC1062 genes at any post-infection time point was calculated using the edgeR package v3.36.0 in R [62]. Genes with false discovery rate (FDR) <0.05, and fold change (FC) ≥2 were considered DEGs. Pelagiphage genes were clustered, and a heatmap of their expression (TPM) was plotted using the pheatmap package v1.0.12 [63] in R. The non-metric multidimensional scaling (NMDS) was performed using the vegan package v2.6-8 in R [64]. The functional annotation of HTCC1062 genes was obtained from the NCBI GenBank database (accession no. CP000084), and KEGG annotation was retrieved from the KEGG online server (https://www.kegg.jp/brite/pub00001.keg). KEGG terms enrichment analysis of DEGs was performed using the clusterProfiler package v4.2.2 in R [65]. KEGG terms with an adjusted *P*-value (*P*.adjust) <0.05 were considered significantly enriched. Linear-regression analysis was performed using R with the lm function to establish the relationship between phage genes and host DEGs based on the TPM, with statistical significance set at *P*-value <0.05 (Table S2). The relationship network between phage genes and host DEGs was visualized using Cytoscape 3.9.1 [66].

## Results and discussion

### Pelagiphages inhibit the growth of HTCC1062 cells

**Figure 1:**
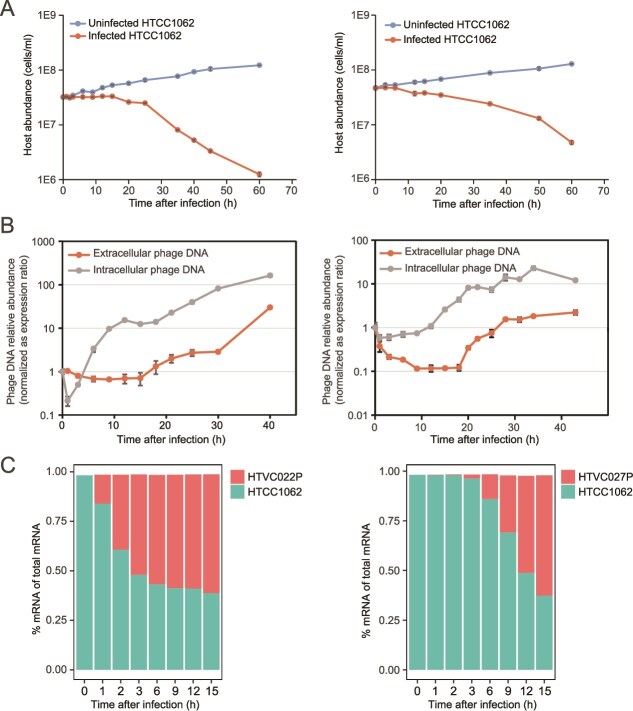
Infection dynamics of two HTCC1062-pelagiphage systems. (A) Growth curves of host HTCC1062 throughout the infection. Left: HTVC022P. Right: HTVC027P. (B) Growth curves of pelagiphages throughout the infection of host HTCC1062. Left: HTVC022P. Right: HTVC027P. The average number of three biological replicates are indicated. The error bars represent the standard deviation of biological triplicates. (C) Ratios of host HTCC1062 and pelagiphages mRNA reads at different time points. Left: HTVC022P. Right: HTVC027P. The ratios were calculated based on the number of reads that mapped phage and host genomes, respectively.

We examined the growth of HTCC1062 under the condition of HTVC022P and HTVC027P infection, respectively. Both pelagiphages inhibited the growth of host cells, with cell densities decreasing sharply after 20 hpi, and >80% of host cells were lysed at 60 hpi (Fig. 1A). Although HTVC022P can integrate into the host genome, the growth curves revealed that it predominantly followed a lytic life cycle. The latent periods of HTVC022P and HTVC027P were ~15 h and 18 h, respectively (Fig. 1B).

### Pelagiphages progressively dominate host transcription

To investigate the transcriptional dynamics of the pelagiphages and the host throughout the course of infection, samples collected at seven different time points (1, 2, 3, 6, 9, 12, 15 hpi) were used for transcriptomic experiments (Fig. 1). Throughout infection of both pelagiphages, phage transcripts gradually increased while host transcripts correspondingly decreased. By 15 hpi, phage transcripts accounted for ~60% of non-rRNA reads, whereas the host transcripts represented <40% (Fig. 1C). A progressive increase of phage transcripts is commonly observed in various host-phage interaction models [11–14, 55, 67, 68]. These results suggest that both HTVC022P and HTVC027P can effectively reprogram host transcription to express phage genes, leading to a gradual transcriptional takeover.

Although both pelagiphages could take over host transcription and reached a similar proportion of transcripts at 15 hpi, HTVC022P initiated this process more rapidly. At 1 hpi, HTVC022P transcripts already accounted for 14.54% of non-rRNA reads (Fig. 1C), whereas HTVC027P transcripts did not increase noticeably until 3 hpi (Fig. 1C). At 1 hpi and 2 hpi, a very limited number of reads (0.15–0.31%) mapped to the HTVC027P genome. A similar delay was also observed during the infection of *Rheinheimera* sp. BAL341 by phage vB_RspM_Barba18A [11]. The mechanism underlying this delayed expression of viral genes remains unclear. This may be due to slow phage attachment, a delayed expression strategy to evade host immune system, or incomplete hijacking of the host’s transcriptional machinery for phage gene expression.

### Temporal transcriptional profiling of pelagiphages

**Figure 2:**
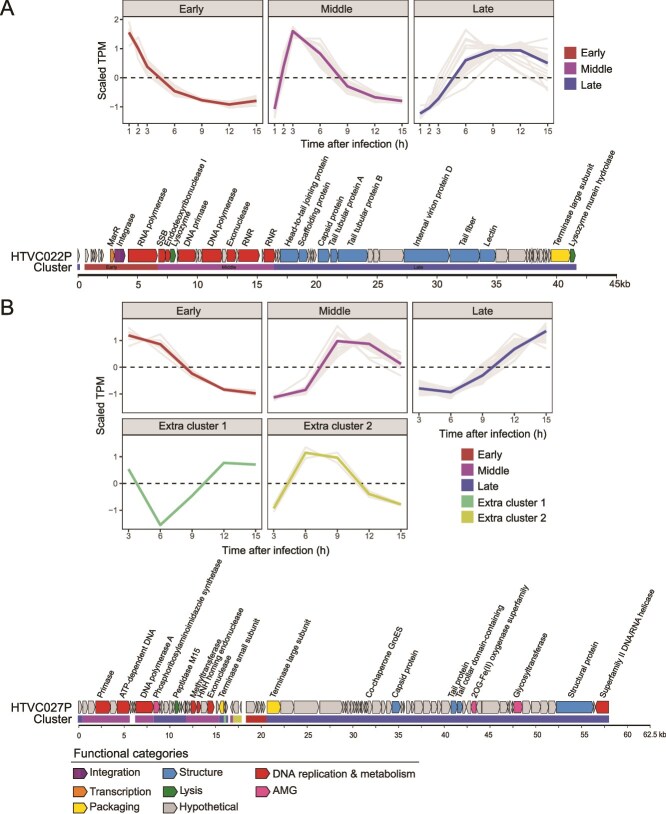
Temporal expression pattern of HTVC022P (A) and HTVC027P genes (B). The TPM values of genes were transformed using z-score over different time points of infection. Early, middle, and late genes are shown in red, purple, and blue, respectively. Genome map of HTVC022P and HTVC027P showing the arrangement of early, middle, and late genes.

RNA-Seq analysis revealed sequential and temporal expression patterns of genes in both pelagiphages (Fig. 2, Fig. S1, and  Table S3). Based on the order in which the transcripts were expressed and peaked, the HTVC022P genes were grouped into three temporal clusters (Fig. 2A, Fig. S1A, and  Table S3). In various phage-host models, phage genes are also typically categorized into three major temporal clusters, including an early cluster associated with host takeover, a middle cluster associated with DNA replication and metabolism, and a late cluster associated with virion morphogenesis and cell lysis [11–13, 15–19]. Among the HTVC027P genes, besides these three typical temporal clusters, two extra temporal clusters were identified (Fig. 2B, Fig. S1B, and  Table S3). Two genes (HTVC027P_gp33 and _gp34) in extra cluster 2 were highly expressed in the early to middle stages (6–9 hpi), and one gene (HTVC027P_gp30) in extra cluster 1 was highly expressed in the early (3 hpi) and late stages (12–15 hpi). The function of HTVC027P_gp33 remains unknown. HTVC027P_gp30 encodes the ribosome alternative rescue factor ArfA, which contains an ArfA (PF03889.18) domain. ArfA is an alternative ribosome rescue factor that facilitates the rescue of stalled ribosomes during translation [69]. Its high expression likely promotes the synthesis of phage proteins. HTVC027P_gp34 contains a Pdase_C33_assoc (PF14756.12) domain. This peptidase C33 may be involved in the processing and maturation of viral polyproteins [70] and also be involved in the deubiquitination process of host proteins, thereby affecting the host’s immune response [71]. Based on these functional annotations, we speculate that both genes are likely involved in viral protein synthesis. The genomic organization of the temporal clusters differed between the two pelagiphages. In HTVC022P, genes within each temporal category were arranged adjacent to each other, whereas in HTVC027P, genes in the three clusters displayed a discontinuous, mosaic-like arrangement. This arrangement of functionally similar genes in HTVC027P may have arisen from horizontal gene transfer or genomic rearrangement. The expression of certain phage-specific genes also revealed distinct infection mechanisms, which may have caused the differences in their infection processes, infection efficiency, and host regulation mechanisms. Two HTVC022P-specific genes, encoding RNAP and transcription factor MarR, were highly expressed in the early stage of HTVC022P infection. In *Escherichia* phage T7, phage-encoded RNAP protein has been reported to facilitate the transcription of phage genes independent of the host transcription system [72]. Similarly, the MarR protein of the *Autographivirales* cyanophage P-SSP7 is considered to be involved in redirecting transcription from the host to the phage [18]. This suggests that HTVC022P likely employs these proteins to transcribe phage genes. The early expression of RNAP and MarR genes may facilitate the rapid takeover of the host transcriptional machinery by HTVC022P. HTVC022P also encodes an integrase gene and has been shown to integrate its genome into the host chromosome at the tRNA-Cys site [46]. The integrase gene was found highly expressed at 1 and 2 hpi, suggesting that HTVC022P integration may occur at the early stage.

In contrast, all early expressed genes of HTVC027P have unknown functions. Considering that phages hijack the host metabolism to transcribe their genes in the early stage of phage infection [18], we hypothesized that these early expressed genes of unknown function in HTVC027P may be crucial for initiating the transcription of phage genes.

### Pelagiphages exert varying effects on HTCC1062 transcription

**Figure 3:**
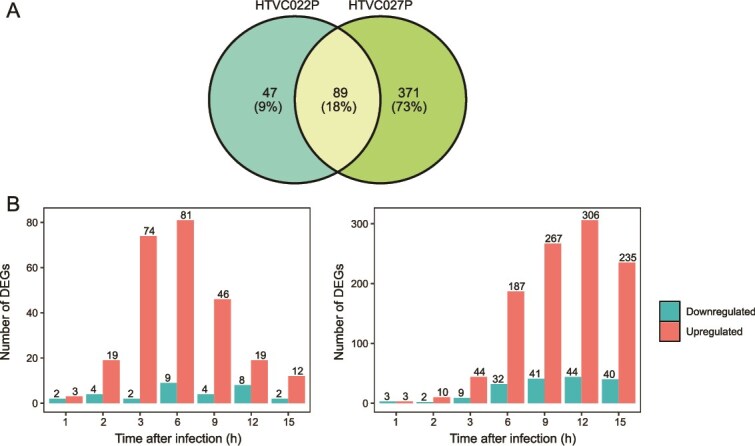
Effect of HTVC022P and HTVC027P infection on host gene expression profile. (A) Venn diagram showing the number of host common and specific DEGs in two pelagiphage–host models. (B) The number of host DEGs across different time points of infection. Left: HTVC022P. Right: HTVC027P.

Although HTVC022P invaded and took over the host machinery more quickly, HTVC027P exerted a greater overall effect on host gene expression. Specifically, 136 host genes (9.81% of non-rRNA genes) were differentially expressed during HTVC022P infection, whereas 460 host genes (33.19%) were differentially expressed during HTVC027P infection (Fig. 3A). During HTVC022P infection, the number of DEGs first increased, reached a maximum at 6 hpi, and then decreased (Fig. 3B). However, during HTVC027P infection, the number of DEGs increased gradually and peaked at 12 hpi. NMDS analysis revealed clear separation of the gene expression patterns of phage-infected host cells at different infection stages in both systems (Fig. S2). These results suggest that these pelagiphages exert distinct effects on host transcription at various infection stages.

**Figure 4:**
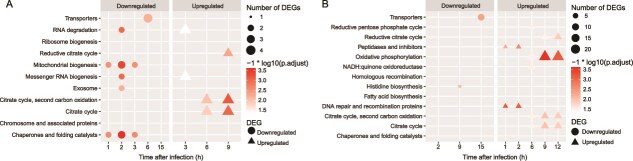
KEGG categories enrichment analysis of host DEGs over each time point of infection. (A) HTVC022P; (B) HTVC027P. KEGG categories with a *P*.adjust <0.05 were considered significantly enriched.

**Figure 5:**
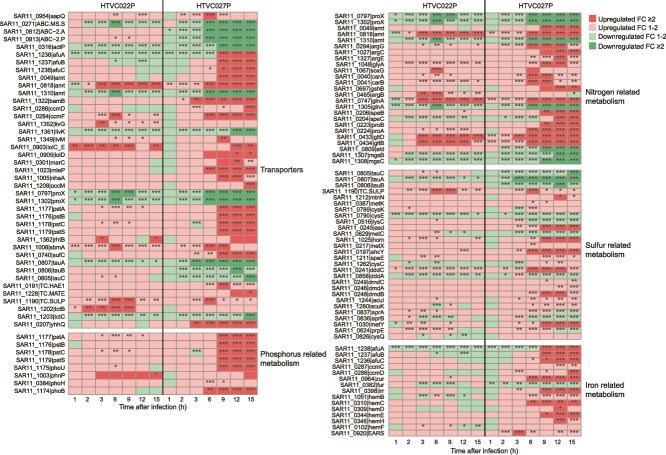
Expression profiles of host DEGs related to the primary nutrient transport and metabolism at each time point of infection. Heatmap plots showing fold change (FC) in the expression of host DEGs. DEGs were arranged according to the function of genes. The significance of changes in gene expression is indicated using an asterisk corresponding to the FDR, (^*^FDR <0.05, ^**^FDR <0.01, ^***^FDR <0.001). Genes with FDR <0.05 and FC ≥2 were considered as DEGs. The abbreviations of the listed genes were provided in Table S5.

In both pelagiphage infections, most DEGs were upregulated, with 88.3% and 83.1% of DEGs being upregulated in HTVC022P and HTVC027P infection, respectively. This is consistent with previous observations in other phage infection systems, in which phages successfully upregulate and redirect the host metabolism to support phage replication [16, 54, 68, 73–75]. Functionally, both pelagiphages enhanced similar core pathways, including DNA replication, repair and recombination, transcription, translation, transporters, peptidases and inhibitors, citrate cycle, pyruvate oxidation, and nitrogen-related metabolism (Figs 4, 5 and Figs S3, S4). However, HTVC027P infection resulted in a more extensive and sustained upregulation of genes within these pathways compared with HTVC022P. Notably, HTVC027P uniquely induced significant enrichment of the oxidative phosphorylation pathway and the quinone oxidoreductase gene (*P*.adjust <.05), neither of which was affected by HTVC022P (Fig. 4). These results indicate that HTVC027P enhanced the host energy production to facilitate viral replication. Furthermore, HTVC027P broadly upregulated genes involved in phosphorus, sulfur, and iron metabolism. In contrast, HTVC022P had minimal impact on these pathways, with only one gene related to phosphorus and sulfur metabolism showing upregulation (Fig. 5). The downregulated host DEGs during both infections were primarily related to transporters, chaperones, and folding catalysts (Fig. 4, Figs S3, and  S4), implying suppression of host protein synthesis that may compromise cellular defense. Together, these results suggest that while both pelagiphage infections lead to the activation of similar host pathways, they employ distinct host takeover mechanisms. HTVC022P employs a faster, more targeted approach, whereas HTVC027P exerts a broader and more sustained transcriptional control. Although HTVC027P takes longer to dominate host transcription, it ultimately reshapes a much larger fraction of host metabolism.

An interaction network analysis revealed extensive and dynamic co-expression relationships between pelagiphages and their hosts, with distinct patterns for each phage (Fig. S5 and Table S2). Specifically, for HTVC022P, most of its early genes showed a positive correlation with host genes involved in transporter and nitrogen-related metabolism. HTVC022P middle genes were predominantly positively associated with host genes involved in translation, DNA metabolism, and central carbohydrate metabolism. The late genes showed positive correlations with host genes involved in central carbohydrate metabolism, DNA metabolism, translation, nitrogen metabolism, as well as chaperones and folding catalysts. For HTVC027P, its early genes positively correlated with host genes related to DNA metabolism, chaperones and folding catalysts, nitrogen-related metabolism, and sulfur-related metabolism. HTVC027P middle and late genes were predominantly positively associated with host genes related to DNA metabolism, transcription, translation, central carbohydrate metabolism, oxidative phosphorylation, iron-related metabolism, nitrogen-related metabolism, and sulfur-related metabolism. Collectively, these results illustrate that pelagiphages progressively and strategically reprogram the host’s transcriptional landscape, with each phage employing a unique molecular approach to hijack host cellular functions.

### Pelagiphages impact the host transcription, DNA replication, repair, and recombination systems

During HTVC022P infection, four host RNAP genes (*rpoB*, *rpoC*, *rpoD*, and *rpoZ*) were upregulated at 2–15 hpi (FC from 1.5 to 1.88) (Fig. S6 and Table S4). Notably, HTVC022P-encoded RNAP was highly expressed at 1 hpi, whereas host RNAPs did not show differential expression at this time point. Whether HTVC022P RNAP is involved in early gene transcription remains to be investigated. In some phage infection systems, such as bacteriophage Xp10 and *Escherichia* phage T7, early viral transcription is dependent on host RNAP, whereas viral RNAP is responsible for the middle and late viral transcription [76, 77]. During HTVC027P infection, five host RNAP genes (*rpoB*, *rpoC*, *rpoD*, *rpoH*, and *rpoZ*) were upregulated throughout the infection (Fig. S6 and Table S4). As HTVC027P does not encode its RNAP gene, it relies on the host’s RNAP to transcribe its genes throughout the infection.

In addition to expressing phage-encoded DNA replication genes, both pelagiphages also caused the upregulation of host DNA polymerase genes (*dnaE*, *dnaN*, *dnaQ*, and *dnaX*) and DNA primase gene (*dnaG*) (Fig. S6 and Table S4). Two phage-encoded ribonucleoside diphosphate reductase genes (*nrdAB*) involved in the conversion of ribonucleotides to deoxyribonucleotides [78] were highly expressed in the middle stage of HTVC022P infection. Although *nrd* genes were not identified in HTVC027P, two host *nrd* genes (*nrdAB*) were highly upregulated (*nrdA*: FC from 3.12 to 8.87; *nrdB*: FC from 2.53 to 5.83) during the middle and late stages (9–15 hpi) of HTVC027P infection (Fig. S6 and Table S4). Overexpression of the host *nrd* genes likely ensured deoxynucleotide production for phage DNA replication. In many phage infection systems, host *nrd* genes were also found to be upregulated [13, 73, 75, 79, 80], suggesting that *nrd* plays an important role in phage DNA replication.

Four host genes comprising the complete SOS response system, including genes encoding the recombination RecA protein (*recA*), LexA transcriptional repressor (*lexA*), and two DNA polymerase V subunits (*umuD* and *umuC*), were upregulated during HTVC027P infection (Fig. S6 and Table S4). The bacterial SOS response system is a constitutively inducible response mechanism that helps cells survive the deleterious consequences of DNA damage [81]. First, RecA forms nucleoprotein filaments with the single-stranded DNA, which can catalyze the self-cleavage of the transcriptional repressor LexA, enabling the expression of the SOS regulon genes. Simultaneously, RecA activates UmuD activity, and the activated UmuD binds to UmuC to form DNA polymerase V, which allows continuous replication across damaged DNA. In addition, the three subunits of excinuclease were also significantly upregulated (FDR <0.05). Excinuclease is an enzyme complex involved in DNA repair to maintain genome stability and integrity by removing damaged or mismatched nucleotides. These results suggest that HTVC027P infection activates the host SOS system, potentially enhancing host survival.

### Effect of pelagiphage infections on host primary nutrient (N, P, and S) metabolism

Both pelagiphage infections affected substrate uptake and export in the host. Specifically, HTVC022P and HTVC027P induced the differential expression of 14 and 38 host transporter genes, respectively (Fig. 5 and Table S4). These transporters are primarily responsible for the transport of small extracellular organic molecules into the cells to provide nutrients [28, 37]. Most differentially expressed transporter genes were upregulated (10 of 14 in HTVC022P infection; 26 of 38 in HTVC027P infection), implying that both pelagiphages activated substance transport in the infected host cells. HTVC027P induced the upregulation of all host high-affinity phosphate transport genes (*pstABCS*). However, HTVC022P did not cause differential expression of the host’s phosphate transport genes. Both pelagiphages upregulated ammonium transporter gene (*amt*) and downregulated the host glycine betaine transporter genes (*proVWX*). In addition, HTVC027P upregulated host iron transporter genes (*afuABC*), whereas HTVC022P downregulated *afuA* and *afuB*. HTVC027P caused the downregulation of three host taurine transport genes (*tauABC*), which were not differentially expressed during HTVC022P infection.

In addition to upregulating the *pstABCS*, HTVC027P also upregulated phosphate regulatory gene (*phoB*) and phosphate transport regulon regulator gene (*phoU*) (Fig. 5 and Table S4). In contrast, HTVC022P infection only induced the upregulation of phosphodiesterase gene *phnp*. This observation indicates that HTVC027P enhanced phosphate metabolism in host cells, while the effect of HTVC022P on host phosphorus metabolism was relatively limited. Under phosphate-limiting conditions, host phosphate uptake genes are regulated by the host’s two-component regulatory system PhoR/PhoB [24, 25]. The upregulation of these genes allows the host to enhance phosphate uptake. This not only supports host survival but also ensures sufficient phosphate to meet the metabolic demands for phage replication and progeny production. This ability to manipulate phosphate uptake and metabolism may therefore explain the higher relative abundance of HTVC027P observed in marine environments [43]. This suggests that the ability of pelagiphages to exploit host phosphate-stress responses represents an important ecological strategy for survival and proliferation in phosphorus-limited oceans.

Regarding nitrogen metabolism, four host genes were significantly upregulated during HTVC022P and HTVC027P infection, including genes encoding glutamine synthetase (*glnA*), glutamate synthase (*gltB* and *gltD*), and ammonium transporter (*amt*) (Fig. 5 and Table S4). Glutamine synthetase catalyzes the conversion of glutamate and ammonia to glutamine, and glutamate synthase catalyzes the conversion of L-glutamine and 2-oxoglutarate to glutamate [82, 83]. Glutamate and glutamine are the primary donors of all nitrogen-containing compounds in the cells. Twelve additional genes related to nitrogen metabolism were upregulated during HTVC027P infection, including genes encoding ammonium transporter (*amt*), glycine hydroxymethyltransferase (*glyA*), glutamate 5-kinase (*proB*), and glutamate-5-semialdehyde dehydrogenase (*proA*) (Fig. 5 and Table S4). These results suggest that both pelagiphages activate the nitrogen uptake and metabolism in the host to meet the nitrogen demand required for phage DNA replication and particle assembly. Similarly, host genes involved in nitrogen metabolism were upregulated in some phage-host models [21]. The upregulation of host genes related to nitrogen transport and metabolism aligns with a previous finding that phages promoted the cellular nitrogen uptake and redirected 75% of the carbon and nitrogen nutrients into viral particles in a *Sulfitobacter*-phage infection system [20].

HTVC027P infection also profoundly impacted host sulfur metabolism. During HTVC027P infection, four genes involved in the cysteine and methionine metabolism pathways were significantly upregulated (Fig. 5 and Table S4). Methionine serves as the sulfur source in the HTCC1062 growth medium. Notably, despite the absence of dimethylsulfoniopropionate (DMSP) in the medium, all genes involved in DMSP demethylation pathway (*dmdABC*) were upregulated. DMSP is an abundant organosulfur molecule in the ocean and is important in the global sulfur cycle and climate regulation [84]. HTCC1062 utilizes DMSP as sulfur source and converted it to methanethiol (MeSH) via the demethylation pathway [32, 34]. MeSH can be catalyzed by cysteine synthase (*metY*) to methionine in HTCC1062, providing a sulfur source to support the growth of HTCC1062. These results indicated that HTVC027P activated the host sulfur metabolism. In contrast, HTVC022P infection exhibited a relatively limited effect on host sulfur metabolism, with no gene involved in the cysteine and methionine metabolic pathways significantly upregulated, and no significant changes observed in genes involved in the DMSP demethylation.

**Figure 6:**
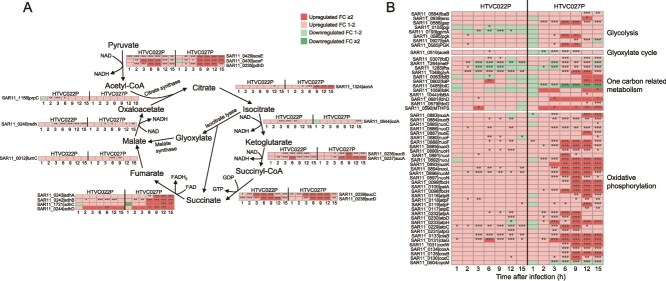
The reprogramming of host energy metabolism during infections. (A) Expression profiles of DEGs associated with the citrate cycle in hosts at each time points. (B) Expression profiles of DEGs associated with glycolysis, one carbon-related metabolism, and oxidative phosphorylation in hosts at each time points. The fold change (FC) values of DEGs expression at each time point of infection were shown in the heatmap. The significance of changes in gene expression is indicated using an asterisk corresponding to FDR (^*^FDR <0.05, ^**^FDR <0.01, ^***^FDR <0.001). Genes with FDR <0.05 and FC ≥2 were considered as DEGs. The abbreviations of the listed genes were provided in Table S5.

The two pelagiphages also diverged in their programming of host iron metabolism. In addition to the upregulated iron transporter genes (*afuABC*), HTVC027P also led to the upregulation of two iron uptake transcriptional regulatory genes (*zur* and *irr*) and five other iron-related genes (Fig. 5 and Table S4), suggesting an increased requirement for iron in host cells. In contrast, two iron transporter genes (*afuAB*) were downregulated during HTVC022P infection. These results suggest that HTVC027P promotes host iron uptake and metabolism, whereas HTVC022P inhibits host iron uptake at certain time points. The upregulation of iron uptake has also been observed in other phage infection systems [55, 73]. Iron is required for cell growth and is a cofactor participating in many metabolic pathways, such as DNA metabolism and bacterial virulence [85]. The upregulation of iron transport genes likely increases intracellular iron concentration, providing essential cofactors for viral replication and thereby enhancing phage replication efficiency. HTVC027P possesses a gene (gp73) encoding 2OG-Fe(II) oxygenase that requires ferrous ions and 2-oxoglutarate (2OG) as cofactors [86]. Enhanced iron metabolism may also provide ATP for viral replication by supporting iron-dependent enzymes within the host respiratory chain, thereby elevating oxidative phosphorylation efficiency. This aligns with our observation of significant upregulation of host genes involved in oxidative phosphorylation. Alternatively, the “Ferrojan Horse Hypothesis” offers another explanation [87]. This hypothesis suggests that phage tail proteins incorporate iron ions, which facilitates phage attachment to host cells. During the synthesis and assembly of phage proteins, iron in the host cell is recycled into the tail proteins of phage progeny. Consistent with this demand, host genes related to iron uptake and metabolism were significantly upregulated at the middle and late stages (Fig. 5 and Table S4). The contrasting expression patterns of iron-related genes in the host observed during the two pelagiphage infections suggest that host iron uptake and metabolism are directly reprogrammed by the phages, rather than being a passive response to phage infection. The elevated intracellular iron content in HTVC027P-infected cells is likely advantageous for phage DNA metabolism and repair, as well as for energy production.

The above divergent strategies may reflect different ecological adaptations of the two pelagiphages. In oligotrophic oceans, the availability of phosphorus, nitrogen, and iron limits primary production and the distribution of phytoplankton biomass [88]. The strategy employed by HTVC027P to enhance host nutrient uptake and metabolism may confer a competitive advantage, facilitating efficient viral replication under nutrient-limiting conditions. Furthermore, such differential viral reprogramming of host nutrient metabolism could also influence biogeochemical cycling and microbial community dynamics in the ocean.

### Energy harvesting pathways in phage-infected HTCC1062 cells

Phage progeny production requires enormous energy. Therefore, we investigated the expression levels of host genes involved in energy production, including the citrate cycle, oxidative phosphorylation, and proteorhodopsin-based phototrophy. Both pelagiphages enhanced the host citrate cycle, with HTVC027P upregulating twice as many DEGs as HTVC022P (Fig. 6A and Table S4). Specifically, HTVC022P upregulated four DEGs involved in the host citrate cycle, whereas HTVC027P upregulated nine DEGs (Fig. 6A and Table S4). Regarding oxidative phosphorylation, 24 phosphorylation-related genes were significantly upregulated in HTVC027P-infected cells, while only one gene was upregulated in HTVC022P-infected cells (Fig. 6B and Table S4).

Although HTCC1062 can utilize glucose as the central carbon source, pyruvate serves as a preferred central carbon source [36]. In the growth medium of HTCC1062, pyruvate was added as a carbon source. During both phage infections, all three genes involved in pyruvate oxidation were significantly upregulated (FDR <0.05) (Fig. 6A and Table S4). This likely increases the supply of acetyl-CoA to the citrate cycle, providing more energy for phage propagation. HTCC1062 has a unique growth requirement [38]. The growth of HTCC1062 does not only rely on pyruvate as the sole carbon; it also requires glycine or glycine precursors, as well as a reduced organosulfur compound such as DMSP or methionine [33, 34, 38].

During HTVC027P infection, a gene encoding glycine hydroxymethyltransferase (*glyA)* was significantly upregulated (FDR <0.05) (Fig. 6B and Table S4). This enzyme catalyzes the transfer of a methylene group between glycine and tetrahydrofolate, generating serine and methylenetetrahydrofolate (methylene-THF) [89]. Methylene-THF can be further oxidized to CO₂, yielding ATP and reduced nucleotides [38]. Genes involved in the THF oxidation pathway, including formate dehydrogenase (*fdhD*) and 5-formyltetrahydrofolate cyclo-ligase (MTHTS), were also upregulated (Fig. 6B and Table S4). This enables the efficient conversion of one-carbon substrates into energy and biosynthetic precursors. Notably, this metabolic capability is a known trait of the host HTCC1062, which has been shown to utilize a variety of one-carbon compounds for energy generation via THF-mediated oxidation [38].

These results demonstrated that both pelagiphages activated the host’s central carbon metabolism, with HTVC027P exerting a more pronounced effect. This finding is consistent with the general role of central carbon metabolism in supplying energy and precursors for phage progeny replication [13, 51, 67]. By enhancing one-carbon metabolism and oxidative phosphorylation, HTVC027P likely enhances the host’s metabolic capacity. This enhanced capacity supports both the host’s survival and the phage’s replication needs, allowing phages to adapt to a wide range of environmental conditions. This also potentially explains the high prevalence of HTVC027P in the ocean.

## Conclusions

This study presents the first transcriptomic investigation into the dynamic interactions between SAR11 and pelagiphages. Our analysis showed that both pelagiphages HTVC022P and HTVC027P effectively reprogram host metabolism to enhance DNA metabolism, transcription, translation, central carbon, and nitrogen metabolism, therefore redirecting host resources toward viral replication. However, they employ distinct strategies. HTVC027P exerted a broader and more sustained transcriptional control, upregulating pathways related to phosphate, sulfur, and iron metabolism, as well as oxidative phosphorylation and one-carbon metabolism. In contrast, HTVC022P adopts a more targeted approach, rapidly hijacking host transcription machinery to prioritize viral gene expression while inducing only minimal changes in these pathways. These findings elucidate the mechanism of pelagiphage infection and the reprogramming of host metabolism, providing valuable insights into SAR11–pelagiphage interactions. Through the differential reprogramming of SAR11 virocells, pelagiphages contribute to the cycling of key elements and exert distinct effects. This highlights the necessity to integrate phage activity into biogeochemical models for a more accurate understanding of marine microbial processes and global biogeochemical cycles.

## Supplementary Material

5_Supplementary_information_ycaf233

Table_S2_ycaf233

Table_S3_phage_express_ycaf233

Table_S4_host_express_ycaf233

Table_S5_Abbreviation_ycaf233

## Data Availability

All RNA-Seq data have been deposited in the NCBI SRA under BioProject accession number PRJNA1253180 and are also available on the figshare (https://doi.org/10.6084/m9.figshare.30632546). The scripts and source data for the figures are available on the figshare (https://doi.org/10.6084/m9.figshare.30632546).

## References

[1] Wommack KE, Colwell RR. Virioplankton: viruses in aquatic ecosystems. *Microbiol Mol Biol Rev* 2000;64:69–114. 10.1128/MMBR.64.1.69-114.2000 10704475 PMC98987

[2] Fuhrman JA . Marine viruses and their biogeochemical and ecological effects. *Nature* 1999;399:541–8. 10.1038/21119 10376593

[3] Suttle CA . Marine viruses—major players in the global ecosystem. *Nat Rev Microbiol* 2007;5:801–12. 10.1038/nrmicro1750 17853907

[4] Paul JH, Sullivan MB, Segall AM. et al. Marine phage genomics. *Comp Biochem Physiol B Biochem Mol Biol* 2002;133:463–76. 10.1016/s1096-4959(02)00168-9 12470812

[5] Hendrix RW, Smith MC, Burns RN. et al. Evolutionary relationships among diverse bacteriophages and prophages: all the world’s a phage. *Proc Natl Acad Sci USA* 1999;96:2192–7. 10.1073/pnas.96.5.2192 10051617 PMC26759

[6] Rohwer F, Thurber RV. Viruses manipulate the marine environment. *Nature* 2009;45:207–12. 10.1038/nature08060 19444207

[7] Brum JR, Sullivan MB. Rising to the challenge: accelerated pace of discovery transforms marine virology. *Nat Rev Microbiol* 2015;13:147–59. 10.1038/nrmicro3404 25639680

[8] Forterre P . Manipulation of cellular syntheses and the nature of viruses: the virocell concept. *C R Chimie* 2011;14:392–9. 10.1016/J.CRCI.2010.06.007

[9] Forterre P . The virocell concept and environmental microbiology. *ISME J* 2013;7:233–6. 10.1038/ismej.2012.110 23038175 PMC3554396

[10] Rosenwasser S, Ziv C, Creveld SGV. et al. Virocell metabolism: metabolic innovations during host-virus interactions in the ocean. *Trends Microbiol* 2016;24:821–32. 10.1016/j.tim.2016.06.006 27395772

[11] Nilsson E, Li K, Hoetzinger M. et al. Nutrient driven transcriptional changes during phage infection in an aquatic *Gammaproteobacterium*. *Environ Microbiol* 2022;24:2270–81. 10.1111/1462-2920.15904 35049095 PMC9305737

[12] Huang S, Sun Y, Zhang S. et al. Temporal transcriptomes of a marine cyanopodovirus and its *Synechococcus* host during infection. *Microbiologyopen* 2021;10:e1150. 10.1002/mbo3.1150 33377630 PMC7885011

[13] Howard-Varona C, Lindback MM, Bastien GE. et al. Phage-specific metabolic reprogramming of virocells. *ISME J* 2020;14:881–95. 10.1038/s41396-019-0580-z 31896786 PMC7082346

[14] Morimoto D, Kimura S, Sako Y. et al. Transcriptome analysis of a bloom-forming cyanobacterium *Microcystis aeruginosa* during Ma-LMM01 phage infection. *Front Microbiol* 2018;9:00002. 10.3389/fmicb.2018.00002 PMC5780444 29403457

[15] Howard-Varona C, Hargreaves KR, Solonenko NE. et al. Multiple mechanisms drive phage infection efficiency in nearly identical hosts. *ISME J* 2018;12:1605–18. 10.1038/s41396-018-0099-8 29568113 PMC5955906

[16] Howard-Varona C, Roux S, Dore H. et al. Regulation of infection efficiency in a globally abundant marine *Bacteriodetes* virus. *ISME J* 2017;11:284–95. 10.1038/ismej.2016.81 27187794 PMC5335546

[17] Doron S, Fedida A, Hernandez-Prieto MA. et al. Transcriptome dynamics of a broad host-range cyanophage and its hosts. *ISME J* 2016;10:1437–55. 10.1038/ismej.2015.210 26623542 PMC5029184

[18] Lindell D, Jaffe JD, Coleman ML. et al. Genome-wide expression dynamics of a marine virus and host reveal features of co-evolution. *Nature* 2007;449:83–6. 10.1038/nature06130 17805294

[19] Moniruzzaman M, Gann ER, Wilhelm SW. Infection by a giant virus (AaV) induces widespread physiological reprogramming in *Aureococcus anophagefferens* CCMP1984 - a harmful bloom algae. *Front Microbiol* 2018;9:752. 10.3389/fmicb.2018.00752 29725322 PMC5917014

[20] Ankrah NY, May AL, Middleton JL. et al. Phage infection of an environmentally relevant marine bacterium alters host metabolism and lysate composition. *ISME J* 2014;8:1089–100. 10.1038/ismej.2013.216 24304672 PMC3996693

[21] Thompson LR, Zeng Q, Chisholm SW. Gene expression patterns during light and dark infection of *Prochlorococcus* by cyanophage. *PLoS One* 2016;11:e0165375. 10.1371/journal.pone.0165375 27788196 PMC5082946

[22] Hurwitz BL, U'Ren JM. Viral metabolic reprogramming in marine ecosystems. *Curr Opin Microbiol* 2016;31:161–8. 10.1016/j.mib.2016.04.002 27088500

[23] Thompson LR, Zeng Q, Kelly L. et al. Phage auxiliary metabolic genes and the redirection of cyanobacterial host carbon metabolism. *Proc Natl Acad Sci USA* 2011;108:757–64. 10.1073/pnas.1102164108 21844365 PMC3182688

[24] Zeng Q, Chisholm SW. Marine viruses exploit their host's two-component regulatory system in response to resource limitation. *Curr Biol* 2012;22:124–8. 10.1016/j.cub.2011.11.055 22244998

[25] Lin X, Ding H, Zeng Q. Transcriptomic response during phage infection of a marine cyanobacterium under phosphorus-limited conditions. *Environ Microbiol* 2016;18:450–60. 10.1111/1462-2920.13104 26522011

[26] Puxty RJ, Evans DJ, Millard AD. et al. Energy limitation of cyanophage development: implications for marine carbon cycling. *ISME J* 2018;12:1273–86. 10.1038/s41396-017-0043-3 29379179 PMC5931967

[27] Giovannoni SJ, Britschgi TB, Moyer CL. et al. Genetic diversity in Sargasso Sea bacterioplankton. *Nature* 1990;345:60–3. 10.1038/345060a0 2330053

[28] Giovannoni SJ . SAR11 bacteria: the most abundant plankton in the oceans. *Annu Rev Mar Sci* 2017;9:231–55. 10.1146/annurev-marine-010814-015934 27687974

[29] Rappe MS, Connon SA, Vergin KL. et al. Cultivation of the ubiquitous SAR11 marine bacterioplankton clade. *Nature* 2002;418:630–3. 10.1146/10.1038/nature00917 12167859

[30] Brown MV, Lauro FM, DeMaere MZ. et al. Global biogeography of SAR11 marine bacteria. *Mol Syst Biol* 2012;8:595. 10.1038/msb.2012.28 22806143 PMC3421443

[31] Grote J, Thrash JC, Huggett MJ. et al. Streamlining and core genome conservation among highly divergent members of the SAR11 clade. *mBio* 2012;3:e00252–12. 10.1128/mBio.00252-12 22991429 PMC3448164

[32] Tripp HJ, Kitner JB, Schwalbach MS. et al. SAR11 marine bacteria require exogenous reduced sulphur for growth. *Nature* 2008;452:741–4. 10.1038/nature06776 18337719

[33] Carini P, Steindler L, Beszteri S. et al. Nutrient requirements for growth of the extreme oligotroph ‘*Candidatus* Pelagibacter ubique’ HTCC1062 on a defined medium. *ISME J* 2013;7:592–602. 10.1038/ismej.2012.122 23096402 PMC3578571

[34] Sun J, Todd JD, Thrash JC. et al. The abundant marine bacterium *Pelagibacter* simultaneously catabolizes dimethylsulfoniopropionate to the gases dimethyl sulfide and methanethiol. *Nat Microbiol* 2016;1:16065. 10.1038/nmicrobiol.2016.65 27573103

[35] Tripp HJ, Schwalbach MS, Meyer MM. et al. Unique glycine-activated riboswitch linked to glycine–serine auxotrophy in SAR11. *Environ Microbiol* 2009;11:230–8. 10.1111/j.1462-2920.2008.01758.x 19125817 PMC2621071

[36] Schwalbach MS, Tripp HJ, Steindler L. et al. The presence of the glycolysis operon in SAR11 genomes is positively correlated with ocean productivity. *Environ Microbiol* 2010;12:490–500. 10.1111/j.1462-2920.2009.02092.x 19889000

[37] Dadon-Pilosof A, Conley KR, Jacobi Y. et al. Surface properties of SAR11 bacteria facilitate grazing avoidance. *Nat Microbiol* 2017;2:1608–15. 10.1038/s41564-017-0030-5 28970475

[38] Sun J, Steindler L, Thrash JC. et al. One carbon metabolism in SAR11 pelagic marine bacteria. *PLoS One* 2011;6:e23973. 10.1371/journal.pone.0023973 21886845 PMC3160333

[39] Carini P, Campbell EO, Morre J. et al. Discovery of a SAR11 growth requirement for thiamin's pyrimidine precursor and its distribution in the Sargasso Sea. *ISME J* 2014;8:1727–38. 10.1038/ismej.2014.61 24781899 PMC4817611

[40] Giovannoni SJ, Tripp HJ, Givan S. et al. Genome streamlining in a cosmopolitan oceanic bacterium. *Science* 2005;309:1242–5. 10.1126/science.1114057 16109880

[41] Tripp HJ . The unique metabolism of SAR11 aquatic bacteria. *J Microbiol* 2013;51:147–53. 10.1007/s12275-013-2671-2 23625213

[42] Zhao Y, Temperton B, Thrash JC. et al. Abundant SAR11 viruses in the ocean. *Nature* 2013;494:357–60. 10.1038/nature11921 23407494

[43] Zhang Z, Qin F, Chen F. et al. Culturing novel and abundant pelagiphages in the ocean. *Environ Microbiol* 2020;23:1145–61. 10.1111/1462-2920.15272 33047445

[44] Du S, Qin F, Zhang Z. et al. Genomic diversity, life strategies and ecology of marine HTVC010P-type pelagiphages. *Microb Genom* 2021;7:000596. 10.1099/mgen.0.000596 34227930 PMC8477408

[45] Buchholz HH, Michelsen ML, Bolanos LM. et al. Efficient dilution-to-extinction isolation of novel virus-host model systems for fastidious heterotrophic bacteria. *ISME J* 2021;15:1585–98. 10.1038/s41396-020-00872-z 33495565 PMC8163748

[46] Zhao Y, Qin F, Zhang R. et al. Pelagiphages in the *podoviridae* family integrate into host genomes. *Environ Microbiol* 2019;21:1989–2001. 10.1111/1462-2920.14487 30474915

[47] Qin F, Du S, Zhang Z. et al. Newly identified HMO-2011-type phages reveal genomic diversity and biogeographic distributions of this marine viral group. *ISME J* 2022;16:1363–75. 10.1038/s41396-021-01183-7 35022515 PMC9038755

[48] Morris RM, Cain KR, Hvorecny KL. et al. Lysogenic host-virus interactions in SAR11 marine bacteria. *Nat Microbiol* 2020;5:1011–5. 10.1038/s41564-020-0725-x 32424337 PMC7387148

[49] Buchholz HH, Bolanos LM, Bell AG. et al. Novel pelagiphage isolate *Polarivirus skadi* is a polar specialist that dominates SAR11-associated bacteriophage communities at high latitudes. *ISME J* 2023;17:1660–70. 10.1038/s41396-023-01466-1 37452097 PMC10504331

[50] Tripp HJ . Counting marine microbes with Guava Easy-Cyte 96 well plate reading flow cytometer. *Protoc Exch* 2008. 10.1038/nprot.2008.29

[51] Brandao A, Pires DP, Coppens L. et al. Differential transcription profiling of the phage LUZ19 infection process in different growth media. *RNA Biol* 2021;18:1778–90. 10.1080/15476286.2020.1870844 33448239 PMC8583145

[52] Zhong Q, Yang L, Li L. et al. Transcriptomic analysis reveals the dependency of *pseudomonas aeruginosa* genes for double-stranded RNA bacteriophage phiYY infection cycle. *iScience* 2020;23:101437. 10.1016/j.isci.2020.101437 32827855 PMC7452160

[53] Wicke L, Ponath F, Coppens L. et al. Introducing differential RNA-seq mapping to track the early infection phase for *Pseudomonas* phage KZ. *RNA Biol* 2021;18:1099–110. 10.1080/15476286.2020.1827785 33103565 PMC8244752

[54] Lood C, Danis-Wlodarczyk K, Blasdel BG. et al. Integrative omics analysis of *Pseudomonas aeruginosa* virus PA5oct highlights the molecular complexity of jumbo phages. *Environ Microbiol* 2020;22:2165–81. 10.1111/1462-2920.14979 32154616 PMC7318152

[55] Blasdel BG, Chevallereau A, Monot M. et al. Comparative transcriptomics analyses reveal the conservation of an ancestral infectious strategy in two bacteriophage genera. *ISME J* 2017;11:1988–96. 10.1038/ismej.2017.63 28498372 PMC5563952

[56] Chevallereau A, Blasdel BG, De Smet J. et al. Next-generation “-omics” approaches reveal a massive alteration of host RNA metabolism during bacteriophage infection of *Pseudomonas aeruginosa*. *PLoS Genet* 2016;12:e1006134. 10.1371/journal.pgen.1006134 27380413 PMC4933390

[57] Li T, Zhang Y, Dong K. et al. Isolation and characterization of the novel phage JD032 and global transcriptomic response during JD032 infection of *clostridioides difficile* ribotype 078. *mSystems* 2020;5:e00017–20. 10.1128/mSystems.00017-20 32371470 PMC7205517

[58] Schmittgen TD, Livak KJ. Analyzing real-time PCR data by the comparative C(T) method. *Nat Protoc* 2008;3:1101–8. 10.1038/nprot.2008.73 18546601

[59] Kopylova E, Noé L, Touzet H. SortMeRNA: fast and accurate filtering of ribosomal RNAs in metatranscriptomic data. *Bioinformatics* 2012;28:3211–7. 10.1093/bioinformatics/bts611 23071270

[60] Chen S, Zhou Y, Chen Y. et al. Fastp: an ultra-fast all-in-one FASTQ preprocessor. *Bioinformatics* 2018;34:884–90. 10.1093/bioinformatics/bty560 30423086 PMC6129281

[61] Liao Y, Smyth GK, Shi W. FeatureCounts: an efficient general purpose program for assigning sequence reads to genomic features. *Bioinformatics* 2014;30:923–30. 10.1093/bioinformatics/btt656 24227677

[62] Robinson MD, McCarthy DJ, Smyth GK. edgeR: a Bioconductor package for differential expression analysis of digital gene expression data. *Bioinformatics* 2010;26:139–40. 10.1093/bioinformatics/btp616 19910308 PMC2796818

[63] Kolde R. pheatmap: pretty heatmaps. 2025. https://github.com/raivokolde/pheatmap.

[64] Oksanen J, Simpson GL, Blanchet FG., et al. vegan: community ecology package. R package version 2.6–8. 2024. https://CRAN.R-project.org/package=vegan.

[65] Wu T, Hu E, Xu S. et al. clusterProfiler 4.0: a universal enrichment tool for interpreting omics data. *Innovation (Camb)* 2021;2:100141. 10.1016/j.xinn.2021.100141 34557778 PMC8454663

[66] Shannon P, Markiel A, Ozier O. et al. Cytoscape: a software environment for integrated models of biomolecular interaction networks. *Genome Res* 2003;13:2498–504. 10.1101/gr.1239303 14597658 PMC403769

[67] Wolfram-Schauerte M, Pozhydaieva N, Viering M. et al. Integrated omics reveal time-resolved insights into T4 phage infection of *E. coli* on proteome and transcriptome levels. *Viruses* 2022;14:2502. 10.3390/v14112502 36423111 PMC9697503

[68] Yang Z, Yin S, Li G. et al. Global transcriptomic analysis of the interactions between phage φAbp1 and extensively drug-resistant *Acinetobacter baumannii*. *mSystems* 2019;4:e00068–19. 10.1128/mSystems.00068-19 31020041 PMC6469957

[69] Keiler KC . Mechanisms of ribosome rescue in bacteria. *Nat Rev Microbiol* 2015;13:285–97. 10.1038/nrmicro3438 25874843

[70] Rawlings ND and Barrett AJ. Introduction: the clans and families of cysteine peptidases. In: Rawlings ND, Salvesen GS (eds.), Handbook of Proteolytic Enzymes. Amsterdam: Elsevier Science, 2013, 1743–73. 10.1016/B978-0-12-382219-2.00404-X

[71] Ozhelvaci F, Steczkiewicz K. Identification and classification of papain-like cysteine proteinases. *J Biol Chem* 2023;299:104801. 10.1016/j.jbc.2023.104801 37164157 PMC10318531

[72] Yang H, Ma Y, Wang Y. et al. Transcription regulation mechanisms of bacteriophages: recent advances and future prospects. *Bioengineered* 2014;5:300–4. 10.4161/bioe.32110 25482231 PMC4156491

[73] Sacher JC, Flint A, Butcher J. et al. Transcriptomic analysis of the *Campylobacter jejuni* response to T4-like phage NCTC 12673 infection. *Viruses* 2018;10:332. 10.3390/v10060332 29914170 PMC6024767

[74] Fan X, Duan X, Tong Y. et al. The global reciprocal reprogramming between mycobacteriophage SWU1 and *Mycobacterium* reveals the molecular strategy of subversion and promotion of phage infection. *Front Microbiol* 2016;7:41. 10.3389/fmicb.2016.00041 26858712 PMC4729954

[75] Furi L, Crawford LA, Rangel-Pineros G. et al. Methylation warfare: interaction of pneumococcal bacteriophages with their host. *J Bacteriol* 2019;201:e00370–19. 10.1128/JB.00370-19 31285240 PMC6755750

[76] Semenova E, Djordjevic M, Shraiman B. et al. The tale of two RNA polymerases: transcription profiling and gene expression strategy of bacteriophage Xp10. *Mol Microbiol* 2005;55:764–77. 10.1111/j.1365-2958.2004.04442.x 15661002

[77] Savalia D, Robins W, Nechaev S. et al. The role of the T7 Gp2 inhibitor of host RNA polymerase in phage development. *J Mol Biol* 2010;402:118–26. 10.1016/j.jmb.2010.07.012 20650282 PMC2941873

[78] Gon S, Faulkner MJ, Beckwith J. *In vivo* requirement for glutaredoxins and thioredoxins in the reduction of the ribonucleotide reductases of *Escherichia coli*. *Antioxid Redox Signal* 2006;8:735–42. 10.1089/ars.2006.8.735 16771665

[79] Li X, Zhang C, Jin X. et al. Temporal transcriptional responses of a vibrio alginolyticus strain to *Podoviridae* phage HH109 revealed by RNA-Seq. *mSystems* 2022;7:e0010622. 10.1128/msystems.00106-22 35400200 PMC9040624

[80] Mojardin L, Salas M. Global transcriptional analysis of virus-host interactions between phage varphi29 and *Bacillus subtilis*. *J Virol* 2016;90:9293–304. 10.1128/JVI.01245-16 27489274 PMC5044823

[81] Maslowska KH, Makiela-Dzbenska K, Fijalkowska IJ. The SOS system: a complex and tightly regulated response to DNA damage. *Environ Mol Mutagen* 2019;60:368–84. 10.1002/em.22267 30447030 PMC6590174

[82] Cornwell EV, MacDonald DW. glnA mutations define the structural gene for glutamine synthetase in *Aspergillus*. *Curr Genet* 1984;8:33–6. 10.1007/BF00405429 24177527

[83] Vanoni MA, Curti B. Glutamate synthase: a complex iron-sulfur flavoprotein. *Cell Mol Life Sci* 1999;55:617–38. 10.1007/s000180050319 10357231 PMC11146855

[84] Galí M, Devred E, Levasseur M. et al. A remote sensing algorithm for planktonic dimethylsulfoniopropionate (DMSP) and an analysis of global patterns. *Remote Sens Environ* 2015;171:171–84. 10.1016/j.rse.2015.10.012

[85] Teh MR, Armitage AE, Drakesmith H. Why cells need iron: a compendium of iron utilisation. *Trends Endocrinol Metab* 2024;35:1026–49. 10.1016/j.tem.2024.04.015 38760200 PMC11616622

[86] Hausinger RP . Fe(II)/α-ketoglutarate-dependent hydroxylases and related enzymes. *Crit Rev Biochem Mol Biol* 2004;39:21–68. 10.1080/10409230490440541 15121720

[87] Bonnain C, Breitbart M, Buck KN. The Ferrojan horse hypothesis: iron-virus interactions in the ocean. *Front Mar Sci* 2016;3:82. 10.3389/fmars.2016.00082

[88] Behrenfeld MJ, O’Malley R, Siegel D. et al. Climate-driven trends in contemporary ocean productivity. *Nature* 2006;444:752–5. 10.1038/nature05317 17151666

[89] Sodolescu A, Sc D, Terradot L. et al. Structural and functional insight into serine hydroxymethyltransferase from *Helicobacter pylori*. *PLoS One* 2018;13:e0208850. 10.1371/journal.pone.0208850 30550583 PMC6294363

