# Supplementary material for: Comparative transcriptomes reveal differential effects on host metabolism reprogramming in two different pelagiphage-SAR11 infection systems
Source: ISME Commun. 2025 Dec 8;5(1):ycaf233. doi: 10.1093/ismeco/ycaf233 (PMC12753301; doi:10.1093/ismeco/ycaf233)
Supplement: 5_Supplementary_information_ycaf233 [file 5_supplementary_information_ycaf233.pdf]

Supplementary information for

Comparative transcriptomes reveal differential effects on host metabolism reprogramming in two different pelagiphage-SAR11 infection systems

Zefeng Zhang[1,#], Xinxin Liu[1,#], Yahui Zhang[1], Hang Xiao[1], Pei Liu[1], Mingyu Yang[1], Fang Qin[1], Ying Wu[1], Hanqi Ying[1], Zuqing Wu[1], Yanlin Zhao[1,2,*]

[1]Fujian Provincial Key Laboratory of Agroecological Processing and Safety Monitoring, College of Juncao Science and Ecology, Fujian Agriculture and Forestry University, Fuzhou, China

[2]Key Laboratory of Marine Biotechnology of Fujian Province, Institute of Oceanology, Fujian Agriculture and Forestry University, Fuzhou, China

[#]These authors contributed equally to this work.

[*]Correspondence: Yanlin Zhao, yanlinzhao@fafu.edu.cn

Supplementary legends

Table S1. The list of specific primers for real-time relative quantitative PCR

Table S2. Results of co-expression analysis of phage genes and host DEGs.

Table S3. Summary of phage gene expression.

Table S4. Summary of host gene expression.

Table S5. The list of abbreviations of host DEGs.

Figure S1. Gene expression heatmaps of HTVC022P and HTVC027P during the infection. The TPM values of genes were normalized using z-score over different time points of infection. Genes were clustered based on by their expression patterns.

Figure S2. non-metric multi-dimensional scaling (NMDS) analysis of host gene expression profiles from different samples at different time points. (A) HTVC022P infected cells; (B) HTVC027P-infected cells. NMDS was performed based on gene expression values TPM.

Figure S3. KEGG categories analysis of host DEGs over each time point during HTVC022P infection. The upregulated and downregulated KEGG categories are colored red and blue, respectively.

Figure S4. KEGG categories analysis of host DEGs over each time point during HTVC027P infection. The upregulated and downregulated KEGG categories are colored red and blue, respectively.

Figure S5. The relationship network between phage genes and host DEGs. (A) HTVC022P; (B) HTVC027P. Phage genes and host genes are shown in triangles and circles, respectively. The red and green edges represent positive and negative correlations, respectively. Early, middle, and late genes are shown in red, purple, and blue, respectively. KEGG categories are shown in different colors.

Figure S6. Effect of pelagiphage infections on genetic information processing at

each time point of infection. Heatmap plots showing fold change (FC) in the expression of host DEGs. DEGs were arranged according to the function of genes. The significance of changes in gene expression is indicated using an asterisk corresponding to the false discovery rate (FDR), (*FDR <0.05, **FDR <0.01, ***FDR <0.001).

Table S1. The list of specific primers for real-time relative quantitative PCR

Name	Sequences
HTVC022P-F	TGGAGTGCTTTATCTGACCGC
HTVC022P-R	GCGTTGCCATACCATCACCA
HTVC027P-F	ACCACCTGGGGTGTGTTTAC
HTVC027P-R	GTTTCAGGGTTCCAGTAGCCA

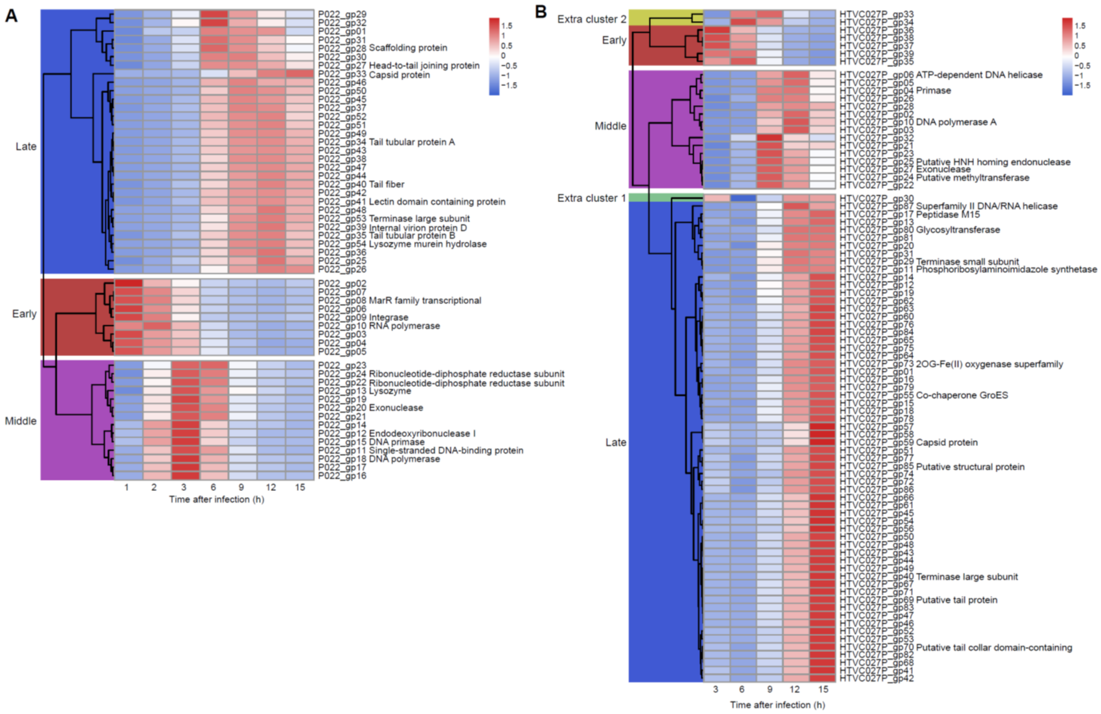

Figure S1. Gene expression heatmaps of HTVC022P and HTVC027P during the infection. The TPM values of genes were normalized using z-score over different time points of infection. Genes were clustered based on by their expression patterns.

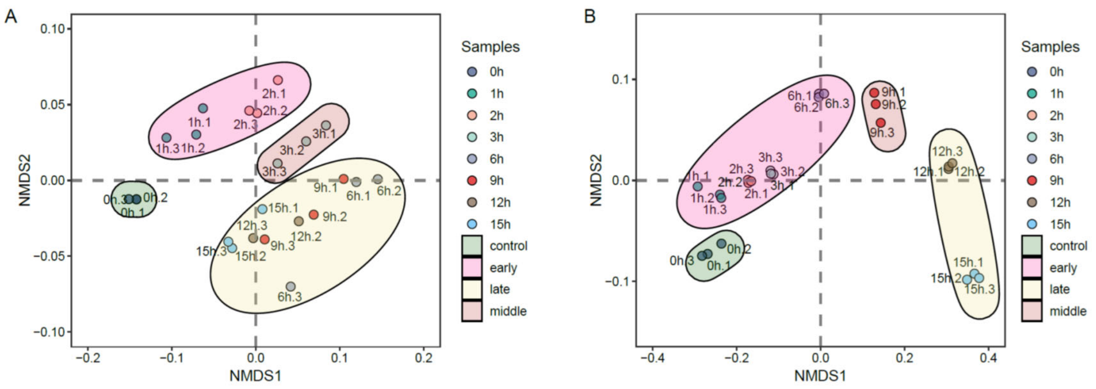

Figure S2. non-metric multi-dimensional scaling (NMDS) analysis of host gene expression profiles from different samples at different time points. (A) HTVC022P infected cells; (B) HTVC027P-infected cells. NMDS was performed based on gene expression values TPM.

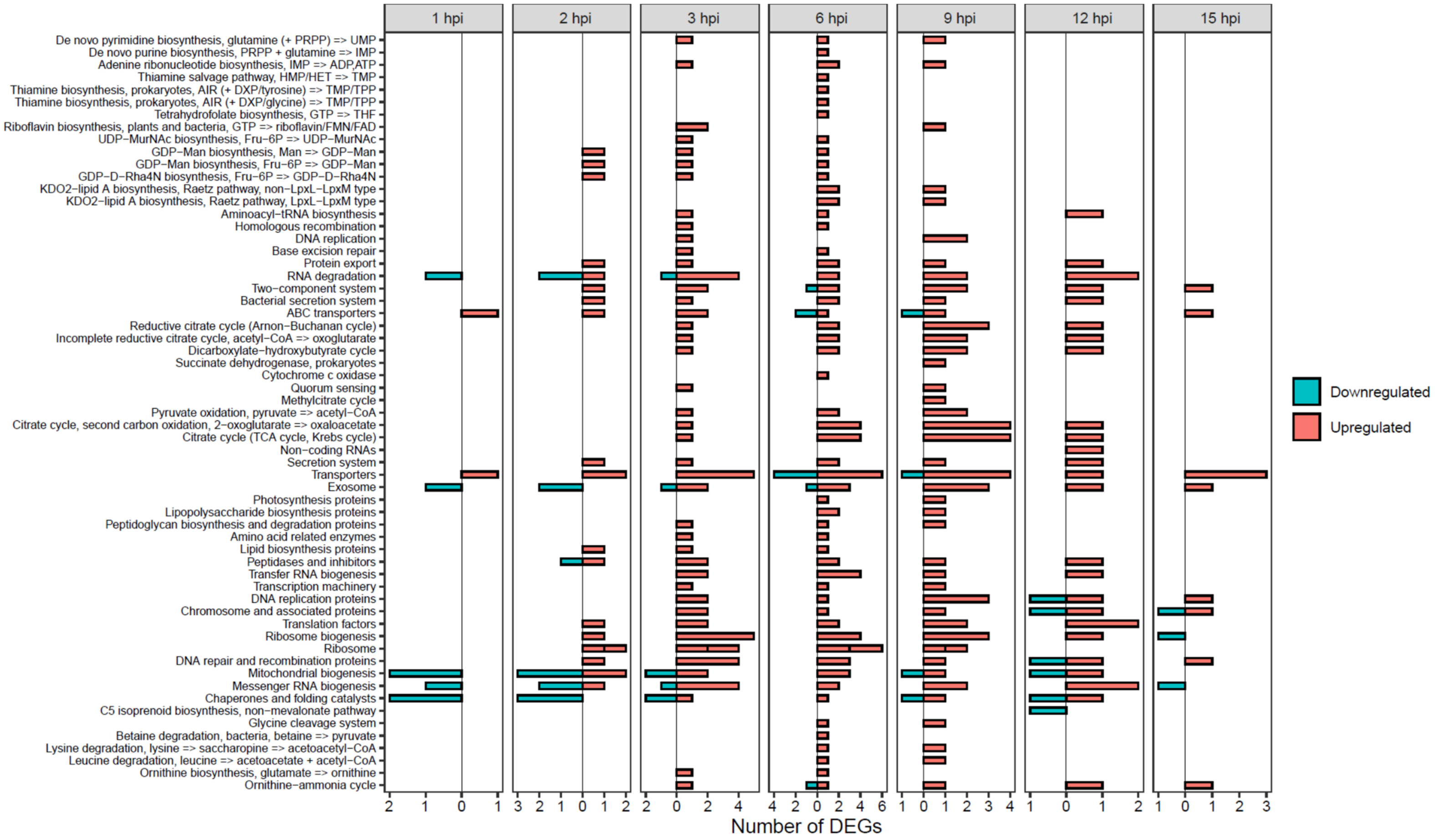

Figure S3. KEGG categories analysis of host DEGs over each time point during HTVC022P infection. The upregulated and downregulated KEGG categories are colored red and blue, respectively.

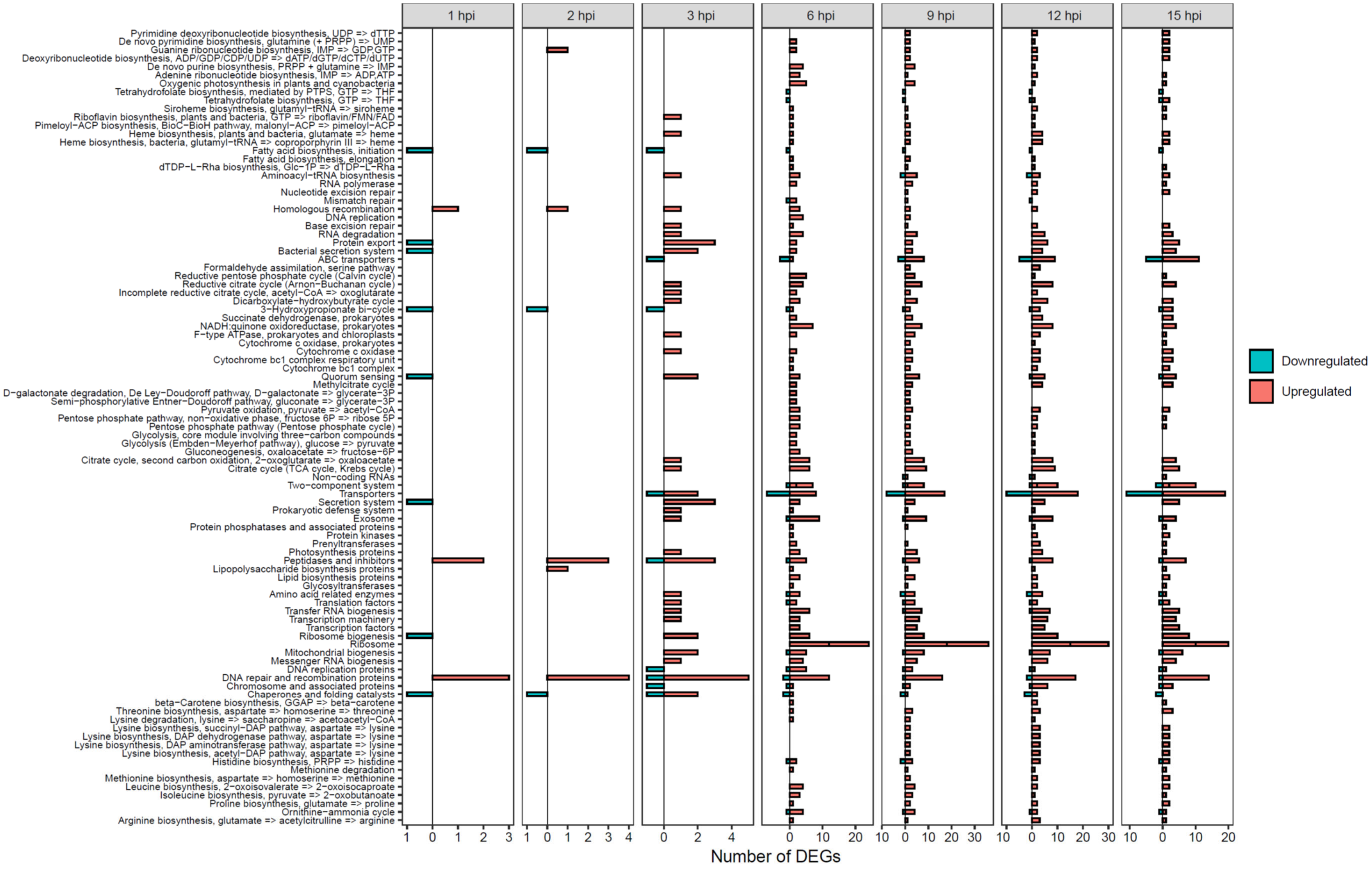

Figure S4. KEGG categories analysis of host DEGs over each time point during HTVC027P infection. The upregulated and downregulated KEGG categories are colored red and blue, respectively.

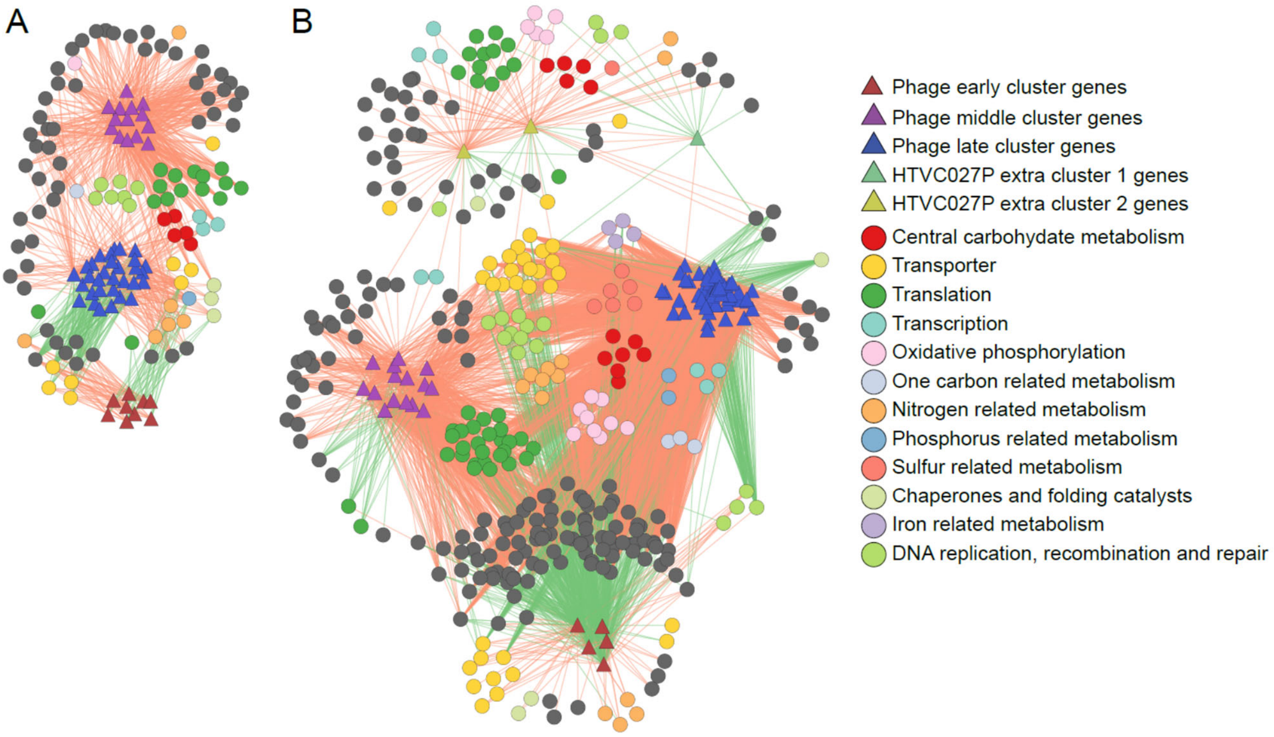

Figure S5. The relationship network between phage genes and host DEGs. (A) HTVC022P; (B) HTVC027P. Phage genes and host genes are shown in triangles and circles, respectively. The red and green edges represent positive and negative correlations, respectively. Early, middle, and late genes are shown in red, purple, and blue, respectively. KEGG categories are shown in different colors.

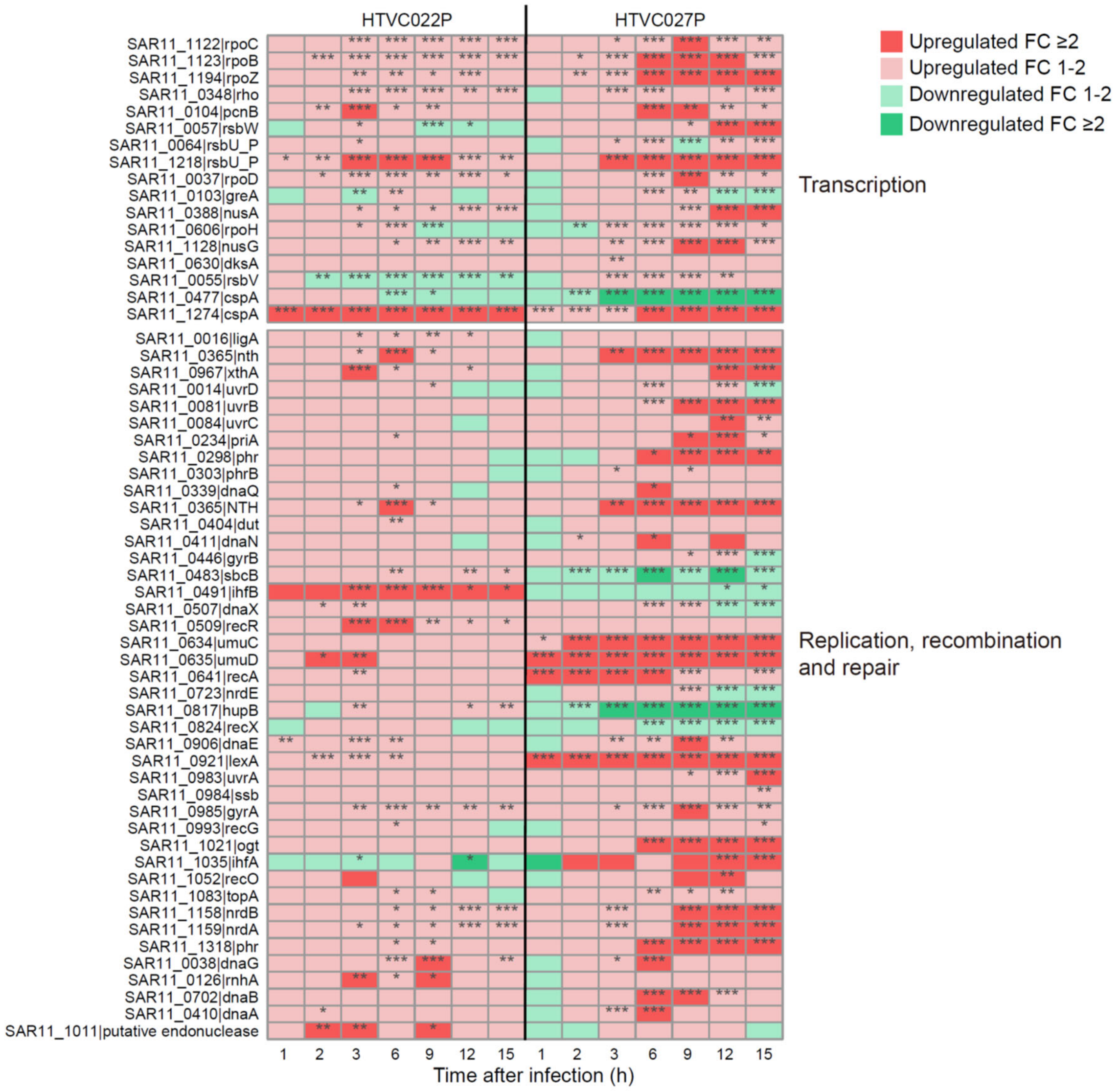

Figure S6. Effect of pelagiphage infections on genetic information processing at each time point of infection. Heatmap plots showing fold change (FC) in the expression of host DEGs. DEGs were arranged according to the function of genes. The significance of changes in gene expression is indicated using an asterisk corresponding to the false discovery rate (FDR), (*FDR <0.05, **FDR <0.01, ***FDR <0.001).
